# Maternal, Decidual, and Neonatal Lymphocyte Composition Is Affected in Pregnant Kidney Transplant Recipients

**DOI:** 10.3389/fimmu.2021.735564

**Published:** 2021-10-28

**Authors:** Dorien Feyaerts, Joshua Gillard, Bram van Cranenbroek, Lina Rigodanzo Marins, Mariam M. S. Baghdady, Gaia Comitini, A. Titia Lely, Henk W. van Hamersvelt, Olivier W. H. van der Heijden, Irma Joosten, Renate G. van der Molen

**Affiliations:** ^1^ Radboud Institute for Molecular Life Sciences, Department of Laboratory Medicine, Laboratory of Medical Immunology, Radboud University Medical Center, Nijmegen, Netherlands; ^2^ Radboud Institute for Molecular Life Sciences, Department of Laboratory Medicine, Laboratory of Medical Immunology, Section Pediatric Infectious Diseases, Radboud University Medical Center, Nijmegen, Netherlands; ^3^ Radboud Center for Infectious Diseases, Radboud University Medical Center, Nijmegen, Netherlands; ^4^ Department of Gynecology and Obstetrics, Hospital de Clinicas de Porto Alegre, Rio Grande do Sul, Brazil; ^5^ Department of Obstetrics and Gynecology, University Medical Center Utrecht, Utrecht, Netherlands; ^6^ Department of Nephrology, Radboud University Medical Center, Nijmegen, Netherlands; ^7^ Department of Obstetrics and Gynaecology, Radboud University Medical Center, Nijmegen, Netherlands

**Keywords:** renal transplantation, neonatal immunity, decidua, uterine immunity, pregnancy, kidney transplantation

## Abstract

Pregnancy after renal transplantation is associated with an increased risk of complications. While a delicately balanced uterine immune system is essential for a successful pregnancy, little is known about the uterine immune environment of pregnant kidney transplant recipients. Moreover, children born to kidney transplant recipients are exposed *in utero* to immunosuppressive drugs, with possible consequences for neonatal outcomes. Here, we defined the effects of kidney transplantation on the immune cell composition during pregnancy with a cohort of kidney transplant recipients as well as healthy controls with uncomplicated pregnancies. Maternal immune cells from peripheral blood were collected during pregnancy as well as from decidua and cord blood obtained after delivery. Multiparameter flow cytometry was used to identify and characterize populations of cells. While systemic immune cell frequencies were altered in kidney transplant patients, immune cell dynamics over the course of pregnancy were largely similar to healthy women. In the decidua of women with a kidney transplant, we observed a decreased frequency of HLA-DR^+^ Treg, particularly in those treated with tacrolimus versus those that were treated with azathioprine next to tacrolimus, or with azathioprine alone. In addition, both the innate and adaptive neonatal immune system of children born to kidney transplant recipients was significantly altered compared to neonates born from uncomplicated pregnancies. Overall, our findings indicate a significant and distinct impact on the maternal systemic, uterine, and neonatal immune cell composition in pregnant kidney transplant recipients, which could have important consequences for the incidence of pregnancy complications, treatment decisions, and the offspring’s health.

## Introduction

Achieving successful pregnancy in women with advanced chronic kidney disease or end-stage renal disease is clinically challenging (1, 2). Renal transplantation is the treatment of choice for most of these patients, especially in women of childbearing age as renal transplantation greatly improves fertility and the ability to conceive (3–5). As a consequence, the number of pregnancies in patients with a kidney transplant is rising (6). Unfortunately, kidney transplant recipients have a higher risk of developing pregnancy complications (5). For instance, preeclampsia occurs in about one-third (21-38%) of the pregnant kidney transplant recipients, while the risk of preeclampsia in the general population is only 3-5% (3, 5–7). Low birth weight children (< 2500 g; 50%) and preterm delivery (< 37 weeks of gestation; 50%) is also more common in kidney transplant recipients compared to the general population (5, 6, 8). These adverse pregnancy outcomes may be a result of impaired (pre-pregnancy) graft function, pre-pregnancy hypertension, or the effect of immunosuppression (5, 9–11). Conversely, delicately balanced immune dynamics, both in the uterus and systemically, are essential for a pregnancy to be successful (12–16). Immune perturbations associated with pregnancy complications are well-documented and may offer an explanation to the higher incidence of complications observed in kidney transplant recipients (16–19).

In addition, the immunosuppressive drug tacrolimus accumulates in the placenta of women with a kidney transplant (20), while azathioprine, tacrolimus, and prednisone are able to cross the placenta and enter the fetal circulation (8). Although the use of kidney transplantation medication has not been directly linked to increased incidence of major congenital malformations (8, 21), limited evidence suggests that *in utero* drug exposure impacts the development of the neonatal immune system. For instance, infants born to kidney transplant recipients had reduced B cell numbers at birth and transplantation itself was associated with an increased risk for hospital admission in the first months of life (22).

In order to investigate the local uterine immune system and systemic immune signatures, we used multiparameter flow cytometry to phenotypically characterize maternal immune cells derived from the decidua after delivery as well as peripheral blood immune cells collected over the course of pregnancy from kidney transplant recipients and healthy individuals with uncomplicated pregnancies. In parallel, we analyzed immune cells in the cord blood of neonates in order to assess the development of the neonatal immune system.

Systemic maternal immune dynamics in kidney transplant recipients largely followed a similar dynamic profile over the course of pregnancy compared to pregnancy in healthy individuals. In addition, we show for the first time that the uterine HLA-DR^+^ regulatory T cell frequencies are affected in women with a kidney transplant, particularly in those treated with tacrolimus. This may suggest that the choice of immunosuppression could influence the risk for the development of complications differently. Compared to healthy controls, reduced regulatory T cell, B cell, NKT-like cell frequencies, and altered monocyte composition was observed at birth in the offspring of kidney transplant recipients, which could have consequences for the offspring’s health outcomes.

## Materials and Methods

### Study Population

19 pregnant women having a healthy, uncomplicated pregnancy (HC) were recruited at the Radboud university medical center, and 14 pregnant women with a kidney transplant (KT) were enrolled at the Radboud university medical center and University Medical Center Utrecht in the Netherlands. 5 KT recipients developed pre-eclampsia during their pregnancy (**Table 1**). Pre-pregnancy kidney function, reported as the average of last 4 measurements before pregnancy, did not differ for those developing pre-eclampsia (**Table 1**). Tacrolimus trough levels did not differ between those receiving “Tacrolimus” or “Tacrolimus+Azathioprine” (data not shown) We collected cord blood (venipuncture of umbilical vein; EDTA tubes), maternal blood during pregnancy, and placentae after delivery. All KT recipients were required to be >1 year post-transplantation with stable graft function before pregnancy could be pursued.

**Table 1:** Donor characteristics.

	Kidney transplant (N=14)	Control (N=19)	
Maternal age (years)	30 (22-38)	31 (26-39)ˆ	
Gestational age at delivery (weeks)	36 (26-38)^‡^	39 (37-41)^‡^	***
Birth weight (g)	2405 (520-3440)^†^	3460 (3150-4503)^†^	***
Pre-eclampsia	5/14	NA	
Mode of delivery			
C-section	6/14	13/19	
Induced+vaginal	4/14	NA	
Induced+C-section	2/14	NA	
Not available	2/14	6/19	
Pre-pregnancy kidney function			
Creatinine (μmol/L)	109.5 (76-188)		
* yes PE*	*116 (95-188)*		ns
* no PE*	*86 (76-157)*		
MDRD-GFR (ml/min/1.73m^2^)	54.5 (26-89)		
* yes PE*	*53 (26-69)*		ns
* no PE*	*68 (36-89)*		
Immunosuppressive drugs			
Azathioprine	7/14	NA	
Tacrolimus	11/14	NA	
* Trough levels (ng/ml)*	*4.5 (4-6.6)*		
Prednisone	12/14	NA	

Median and range (min to max) are shown for age of mother, gestational age, birth weight, creatinine, MDRD-GFR (glomerular filtration rate from Modification of Diet in Renal Disease equation), and tacrolimus trough levels. Tacrolimus trough levels closest prior to delivery are reported.

ˆInformation not available for 5 mothers. ^‡^Information not available for 1 and 4 pregnancies, respectively. ^†^Information not available for 1 out of 14 and 10 out of 19 deliveries, respectively. ***p-value < 0.001, ns, not significant; Mann-Whitney test. PE, pre-eclampsia; NA, not applicable.

First trimester material – as used in *in vitro* cultures – was obtained from discarded uterine tissue after elective pregnancy termination, upon written consent. No further clinical information was obtained from these donors.

This study was approved by the local review board (Commissie Mensgebonden onderzoek region Arnhem-Nijmegen; CMO nr. 2014-232 and CMO nr. 2017-3253). In accordance with the Dutch Medical Research Involving Human Subject Act (WMO), all participants provided written informed consent before material was donated and included in this study.

### Isolation of Lymphocytes From Maternal Blood and Cord Blood

One ml of blood was lysed with 25 ml lysis buffer (NH_4_CL + KHCO_3_/Na_4_EDTA diluted in H_2_O) for 10 minutes and washed 3x times with PBS. Lysed blood samples were used when only surface staining was performed. For intracellular staining protocol, lymphocytes were isolated by density gradient centrifugation (Lymphoprep, Axis-Shield PoC AS). After centrifugation (801 x g, 15 minutes, no brake), the lymphocyte layer was collected and isolated cells were washed twice with PBS before further analysis.

### Isolation of Uterine Lymphocytes

Decidua parietalis was collected from the obtained term placentae as previously described (13). After removing the amnion, the decidua parietalis (i.e. maternal layer of the placental membranes surrounding the fetus) was carefully scraped from the chorionic trophoblast layer. First trimester decidual tissue was separated from villous tissue. The tissue was washed thoroughly with PBS, minced with scissors and washed again until the supernatant became clear. The tissue was incubated with 0.2% collagenase (Gibco Life Technologies) and 0.04% DNAse (Roche Diagnostics) in a water bath at 37°C while shaking. After 60 minutes, digested tissue was washed with supplemented RPMI (RPMI 1640 medium supplemented with 1 mM pyruvate, 2 mM glutamax, 100 U/ml penicillin, and 100 mg/ml streptomycin; Thermo Fischer) and passed through a 100 µm, 70 µm, and 40 µm cell strainer (Greiner). Lymphocytes were obtained after density gradient centrifugation (801 x g for 15 minutes, no brake) on a discontinuous Percoll gradient (1,050 g/ml, 1,056 g/ml and 1,084 g/ml; GE Healthcare). Lymphocytes were isolated from the 1,084-1,056 g/ml interface.

### *In Vitro* Stimulation of First Trimester Uterine Lymphocytes

100.000 freshly isolated uterine lymphocytes were cultured with anti-CD3/CD28 microbead stimulation (1:2 bead-to-cell ratio) in the absence or presence of azathioprine (6-mercaptopurine; active metabolite of azathioprine) or tacrolimus (FK506), in 10% HPS culture media (RPMI 1640 medium supplemented with 1 mM pyruvate, 2 mM glutamax, 100 U/ml penicillin, 100 mg/ml streptomycin, and 10% Human Pooled Serum) in a 96-well U-bottom plate for 5 days at 37°C in a humidified 5% CO_2_ incubator. Azathioprine and tacrolimus concentrations were chosen based on *in vivo* serum levels. Serum levels for mercaptopurine (azathioprine) range 235-500 pmol per 8x10^8^ red blood cells (communication with nephrologist) in adult renal transplant patients after oral administration, with a maximum serum concentration of 50 ng/ml after oral administration (23). Tacrolimus serum levels range 5-6 ng/ml (communication with nephrologist) in adult renal transplant patients, with serum concentration of 5-15 ng/ml reported during pregnancy (24). The influence of these immunosuppressive drugs on cytokine expression and regulatory T cell frequency was assessed by flow cytometry.

### Flow Cytometric Analysis

**Supplemental Table 1** lists the fluorochrome-conjugated monoclonal antibodies that were used to phenotypically characterize immune cells in cord blood, peripheral blood, uterine samples, and *in vitro* assays. Samples were analyzed on a 10-color Navios™ flow cytometer (480 nm argon blue laser, 405 nm solid state violet laser, 636 nm solid state laser, Beckman Coulter). In brief, cells were washed twice with PBS-0.2%BSA (bovine serum albumin, Sigma-Aldrich) before staining with surface antibodies for 20 minutes at room temperature, protected from light. After permeabilization and fixation, intracellular staining was performed for 30 minutes at 4°C in the dark. For intracellular cytokine expression, cells were first stimulated for 4 hours with PMA (phorbol-12-myristate-13-acetate; 12.5 ng/ml), ionomycin (500 ng/ml), and brefeldin A (5 μg/ml) at 37°C in a humidified 5% CO_2_ incubator. IFN-γ and IL-17 were used as proxy cytokines for pro-inflammatory Th1 and Th17 cell subsets respectively. 28 immune cell subpopulations (**Figure S1**) were identified in uterine, peripheral blood, and cord blood samples by manual gating using Kaluza software v2.1 (Beckman Coulter). The gating strategy is illustrated in **Figure S1**.

### Statistical Analysis

GraphPad Prism was used to perform statistical analysis. For comparisons of 2 groups, non-parametric Mann-Whitney test was used to compare immune cell subsets in maternal blood, decidua, and blood of the control (HC) and transplanted group (KT). For comparison of multiple groups, non-parametric Kruskal-Wallis test was used, where p-values were calculated against the HC group with a post-hoc Dunn test. A simple linear regression was performed for data in **Figures S3A, S8A** to test whether the slope of the regression lines is significantly different. P-values < 0.05 are considered significant. Boxplots and percentages in text are depicted as median with [interquartile range].

## Results

### Decidual-Derived HLA-DR^+^ Regulatory T Cells Are Decreased in Kidney Transplant Recipients

To investigate the local uterine immune environment in women with a kidney transplant, we collected the decidua parietalis from placentae (after delivery) of kidney transplant recipients and healthy, uncomplicated pregnancies. The frequency of monocytes, NK cells, B cells/subsets, T cells/subsets [regulatory T cells (Treg), effector/memory T cells (CD45RA and CCR7 expression (13)], and cytokine expressing T cells (IFN-γ and IL-17) was examined by multiparameter flow cytometry (**Figure S1A**: gating strategy and assessed immune cell subpopulations).

**Figure 1:**
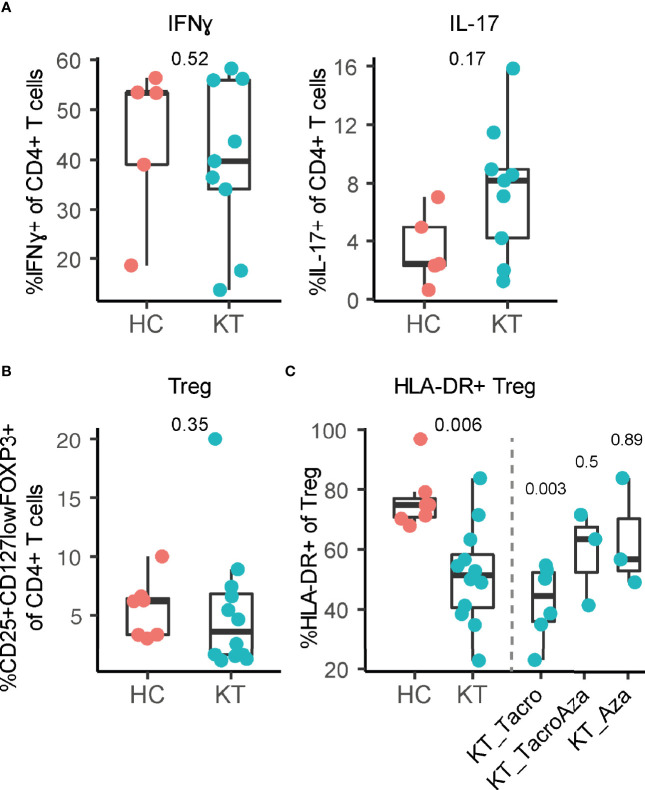
Frequency of HLA-DR^+^ regulatory T cells is affected in decidua of kidney transplant recipients (KT). **(A)** Percentage IFN-γ^+^ and IL-17^+^ CD4^+^ T cells, **(B)** percentage CD25^+^CD127^low^FOXP3^+^ regulatory T cells (Treg) and **(C)** percentage HLA-DR^+^ Treg in decidua from KT and healthy individuals (HC) are shown. Percentage HLA-DR^+^ Treg is separated based on which combination of tacrolimus (Tacro) and azathioprine (Aza) is used.

We observed no difference in frequency for monocytes, NK cells, T cell subsets, and B cell subsets in the decidua of kidney transplant recipients compared to healthy individuals (**Figure S2**). In addition, no difference in percentage IFN-γ^+^ and IL-17^+^ CD4^+^ T cells in the decidua was observed, representing pro-inflammatory Th1 and Th17 cells respectively (**Figure 1A**). While we did not observe a difference in CD25^+^CD127^low^FOXP3^+^ Treg frequency (**Figure 1B**), we did observe a significant decrease in the percentage of HLA-DR^+^ Treg within the total uterine Treg pool of kidney transplant recipients compared to healthy individuals (**Figure 1C**). Importantly, to exclude that this difference was due to a difference in gestational age at delivery (**Table 1**), HLA-DR^+^ Treg frequency was plotted against gestational age at delivery with a linear regression (**Figure S3A**). This showed that no significant difference was observed for the linear regression slopes, indicating that uterine HLA-DR+ Treg frequencies in kidney transplant recipients behave similarly over the course of pregnancy. This suggests that the decreased frequency is inherent to the kidney transplant environment, and likely not due to a difference in gestational age. Interestingly, the greatest decrease in percentage HLA-DR^+^ Treg could be observed for those kidney transplant recipients that used tacrolimus (without azathioprine); 44.2 [21.4]% versus 74.8 [8.9]% in controls (**Figure 1C**). Interestingly, this decrease in HLA-DR^+^ Treg was less when azathioprine was added to the drug regimen (63.3 [30.2]%), or when tacrolimus was not used at all (56.2 [34.8]%; azathioprine only). In addition, the bimodal distribution observed for NK, T, and B cell frequencies in **Figure S2** is likely due to a difference in drug regimen as well (**Figure S3B**). This shows that different immunosuppressive drugs could have distinct effects on uterine immunity.

Pregnancy complications such as preeclampsia are considered a consequence of defective placentation during the first weeks of pregnancy (25). Due to ethical constraints, it is not feasible to assess the *in vivo* effect of different immunosuppressive drugs on the first trimester uterine immune environment. Moreover, studies conducted with peripheral cells cannot be extrapolated completely to the uterine environment due to the clear difference in immune cell composition and function (13, 15). Therefore, we cultured uterine immune cells isolated from first trimester decidual tissue in the presence of tacrolimus or azathioprine, at concentrations that resemble *in vivo* serum levels (see Methods). While azathioprine did not influence T cell cytokine expression, diminished cytokine expression could be observed in decidual T cells cultured in the presence of tacrolimus (**Figure S4A**). Frequency of FOXP3^+^CD127^low^CD4^+^ Treg was diminished by both azathioprine and tacrolimus after *in vitro* culture (**Figure S4B**).

Overall, we showed that kidney transplant recipients have lower frequencies of uterine HLA-DR^+^ Treg upon delivery, predominantly when tacrolimus is used, and that *in vitro* exposure of uterine immune cells to immunosuppressive drugs affected Treg frequency and T cell cytokine expression.

### Maternal Systemic Immunity in Pregnant Kidney Transplant Recipients Follow Similar Dynamic Profiles Compared to Healthy Controls

Systemic immune signatures can be observed over the course of pregnancy (12, 26) and changing signatures are associated with pregnancy complications (27). In kidney transplant recipients, it is unclear how systemic maternal immune dynamics change during pregnancy. Immune cell frequencies were assessed by multiparameter flow cytometry (**Figure S1B**: gating strategy and assessed immune cell subpopulations) in peripheral blood samples collected over the course of pregnancy. To assess dynamic changes in systemic immunity over the course of pregnancy, the relationship between cell frequency and gestational age at time of sample collection was plotted with a LOESS regression.

**Figure 2:**
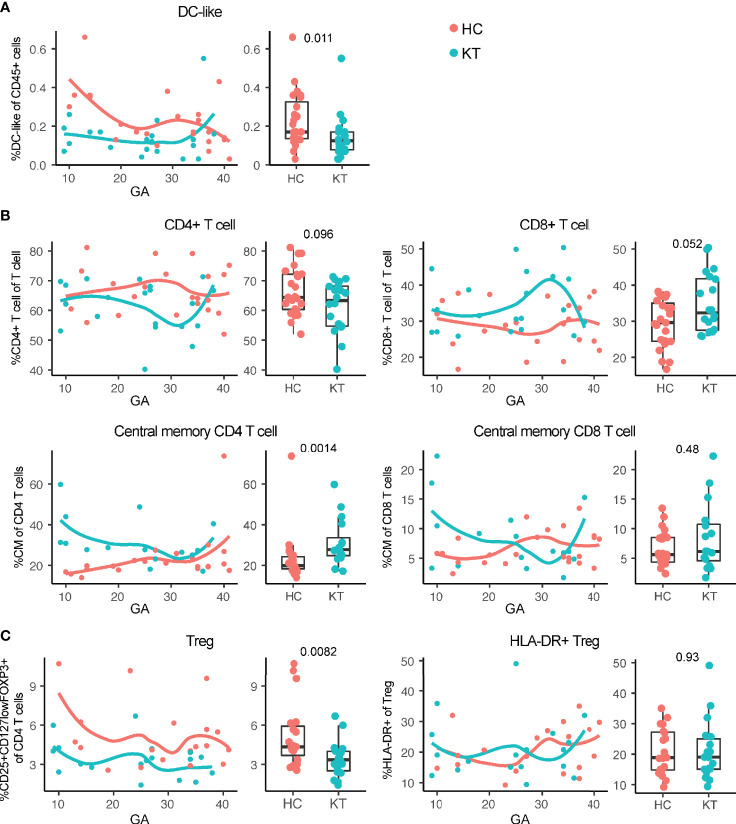
Maternal systemic immunity is affected in pregnant kidney transplant recipients (KT). **(A)** Percentage DC-like cells (CD45^+^CD19^-^CD3^-^CD56^-^ CD16^-^CD14^low^ HLA-DR^+^), **(B)** percentage CD4^+^ T cell, CD8^+^ T cell, and central memory (CM; CD45RA^−^CCR7^+^) CD4 and CD8 T cells, and **(C)** percentage regulatory T cells and HLA-DR^+^ Treg in peripheral blood from KT and healthy individuals (HC) are shown. Frequencies of peripheral blood immune cells are depicted both in boxplots (median + interquartile range) and as regression (LOESS) with gestational age (GA) at time of sample collection.

**Figure 3:**
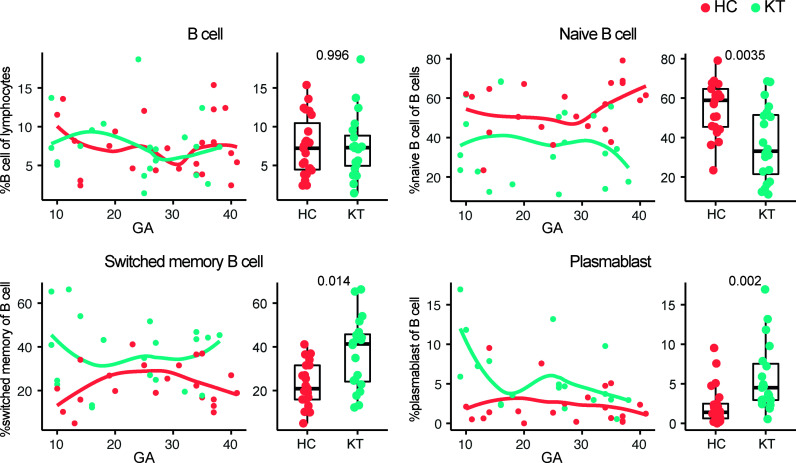
Systemic B cell frequencies are affected in pregnant kidney transplant recipients (KT). Percentage B cells, naïve B cells (CD27^-^IgD^+^), switched memory B cells (CD27^+^IgD^-^), and plasmablasts (CD24^+^IgD^-^CD38+) in peripheral blood from KT and healthy individuals (HC) are shown. Frequencies of peripheral blood immune cells are depicted both in boxplots (median + interquartile range) and as regression (LOESS) with gestational age (GA) at time of sample collection.

Monocytes, NK cells, several T cell subset frequencies, and IFN-γ^+^ and IL-17^+^ CD4^+^ T cells in peripheral blood of pregnant women with a kidney transplant followed similar immune dynamic changes over the course of pregnancy as compared to healthy individuals (**Figure S5**), while slightly different maternal immune dynamics could be observed for CD4 and CD8 (central memory) T cells, and DC-like cells (**Figure 2**). In addition, combining frequencies from all gestational ages into a discrete variable showed that the overall frequency of CD25^+^CD127^low^FOXP3^+^ Treg was consistently lower in kidney transplant recipients compared to controls (**Figure 2C**), in accordance with numerous observations in peripheral blood of kidney transplant patients receiving immunosuppression, especially when receiving calcineurin inhibitors (28–31). The decreased frequency of HLA-DR^+^ Treg observed in decidua was not reflected in the mother’s peripheral blood (**Figure 2C**). In addition, the observation in the decidua that tacrolimus use showed the greatest decrease in percentage HLA-DR^+^ Treg was not reflected in maternal blood either (**Figure S6**). Tacrolimus (without azathioprine) did however showed the greatest decrease in percentage of Treg (**Figure S6**). While B cell subset immune dynamics did not differ significantly over the course of pregnancy, a decreased percentage of naïve B cells and increased percentage of plasmablast and switched memory B cells could be observed overall in peripheral blood of pregnant kidney transplant recipients compared to controls (**Figure 3**), suggesting a switch in B cell phenotype from naïve to a more memory phenotype in pregnant women with a kidney transplant.

Overall, results showed that while the systemic immune system of pregnant kidney transplant recipients is different compared to uncomplicated and healthy pregnancies, the immune cell changes associated with pregnancy progression largely followed the same dynamic profile.

### Immunosuppressive Drug Use During Pregnancy Affects the Neonatal Immune System

In kidney transplant recipients, neonatal immune development occurs under immunosuppressive drug exposure (8, 32–34). To assess whether being born to a mother with a kidney transplant influences the development of the neonatal immune system, we collected cord blood (umbilical vein) of neonates born to kidney transplant recipients and to healthy, uncomplicated pregnancies. Similar to the maternal blood and decidual phenotyping, the neonatal immune cell composition was characterized using multiparameter flow cytometry (**Figure S1C**).

**Figure 4:**
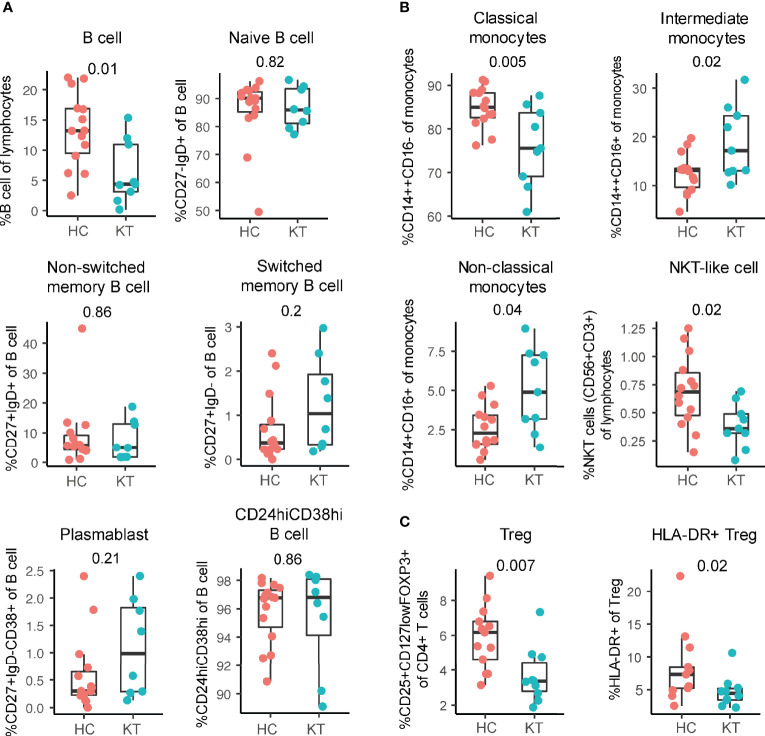
Innate immunity is affected in neonates born to pregnant kidney transplant recipients (KT). **(A)** Percentage of total B cells and B cell subsets. B cell subsets: naïve (CD27^-^IgD^+^), plasmablast (CD24^+^IgD^-^CD38+), non-switched memory (CD27^+^IgD^+^, switched memory (CD27^+^IgD^-^), and CD24^h^iCD38^hi^ in cord blood of neonates born to KT and HC are shown as a percentage of B cells. **(B)** Percentage classical (CD14^++^CD16^-^), intermediate (CD14^++^CD16^+^), non-classical (CD14^+^CD16^+^) monocytes, and NKT-like cells in cord blood of neonates born to KT and healthy individuals (HC) are shown. **(C)** Percentage of regulatory T cells (Treg) and HLA-DR^+^ Treg in cord blood of neonates born to KT and HC are shown.

In comparison to newborns of mothers with a healthy and uncomplicated pregnancy, cord blood of neonates born to kidney transplant recipients showed decreased B cell, Treg, and HLA-DR^+^ Treg frequencies with no difference in percentage IFN-γ^+^ and IL-17^+^ CD4^+^ T cells (**Figure 4** and **Figure S7**), complementing prior reports (22, 35–39). This decreased percentage of Treg in the neonates born to kidney transplant recipients – who are largely born prematurely (**Table 1**) – is in contrast to literature where neonates born earlier show increased Treg frequencies that decrease with advancing gestational age (40, 41). When plotting Treg frequencies according to gestational age, we indeed observed decreasing Treg frequencies with advancing gestational age in both group (**Figure S8A**). This suggests that the *in utero* environment might have had an influence on the development of Treg. In addition, similarly to our results in the decidua, the strongest decrease in HLA-DR^+^ Treg percentages in cord blood was observed when tacrolimus, with or without azathioprine, was used during pregnancy (**Figure S8B**).

While B cell frequencies were decreased, in-depth B cell phenotyping performed here showed that the composition of the B cell population did not differ between our two groups of infants; i.e. no difference in naive, non-switched, switched, plasmablast, and CD24^hi^CD38^hi^ B cell frequencies were observed at birth (**Figure 4A**), suggesting there is an overall B cell decrease rather than a decrease of a specific subset.

In addition, NKT-like cells and classical monocyte (CD14^++^CD16^-^) percentages were significantly decreased and non-classical monocytes (CD14^+^CD16^+^) and intermediate monocytes (CD14^++^CD16^+^) monocytes increased in cord blood of children born to kidney transplant recipients compared to infants born to women with healthy and uncomplicated pregnancies (**Figure 4B**). This shows that next to the adaptive immune system, also the innate system is affected at birth in neonates born to kidney transplant recipients.

## Discussion

Renal transplantation greatly restores fertility in women with chronic kidney or end-stage renal disease (1–5). However, a higher risk of pregnancy complications can be observed in women with a kidney transplant (3, 5–8). Here, we provide an overview of the immune cell characteristics of the maternal systemic and uterine immune environment of women with a kidney transplant and healthy individuals with uncomplicated pregnancies, and the neonatal immune system of their offspring. We showed decreased frequencies of HLA-DR^+^ Treg in the decidua of women with a kidney transplant, particularly in those treated with tacrolimus. While systemic immune cell frequencies were altered in kidney transplant patients, immune cell dynamics over the course of pregnancy were largely similar to healthy women. In addition, we report that the neonatal immune system at birth is affected in the offspring of kidney transplant recipients.

To mediate suppression against fetus-specific antigens, Treg with a suppressive phenotype are enriched in the decidua during healthy pregnancy (13, 42–46). HLA-DR^+^ Treg have been shown to be highly suppressive (47, 48) and here, we found decreased frequencies of HLA-DR^+^ Treg in decidual tissue after delivery of women with a kidney transplant. This decreased frequency is likely not due to a difference in gestational age at delivery. Systemically, reduced Treg frequencies could also be observed during pregnancy in kidney transplant recipients. Pregnancy complications such as preterm labor, are associated with altered Treg populations and disturbed Treg tolerance (46, 49, 50). This may suggest there is a loss of fetal tolerance at the maternal-fetal interface in women with a kidney transplant, which could explain the observed incidence of pregnancy complications such as preterm birth in these patients (5, 6, 8). Moreover, the strongest decrease in uterine HLA-DR^+^ Treg and maternal blood Treg was observed in those women who were prescribed tacrolimus, in comparison to those who used azathioprine (only azathioprine or azathioprine in combination with tacrolimus). This suggests that azathioprine may have a less detrimental impact on the uterine and systemic immune system than tacrolimus and perhaps could be more favorable to use during pregnancy. A large retrospective study conducted in the Netherlands indeed showed a trend towards lower birthweight (< 2500 grams) with a high rate of preterm delivery in pregnant kidney transplant recipients with a calcineurin inhibitor-based regimen (e.g. tacrolimus) (51) (unpublished manuscript). In our *in vitro* culture of first trimester uterine lymphocytes, both drugs reduced the frequency of Treg but only tacrolimus diminished cytokine expression by T cells, again potentially suggesting that azathioprine might have a less detrimental impact on the uterine system.

Dynamic changes in immune signatures can be observed in maternal peripheral blood over the course of pregnancy (12, 26). Here, we observed that these dynamics largely progressed in the same manner for women with a kidney transplant compared to controls. Only CD4^+^ and CD8^+^ (central memory) T cells, and DC-like cells frequencies progressed differently. This suggests that the majority of normal pregnancy associated frequency changes are not affected in kidney transplant recipients. However, it would be interesting to assess whether changes in signaling and functional responses could be affected. In addition, we did observe clear differences in the overall frequency of peripheral immune cells such as a change in B cell phenotype from naïve to a more memory phenotype (plasmablast and switched memory B cell) in pregnant women with a kidney transplant compared to women with uncomplicated pregnancies. Although calcineurin inhibitors such as tacrolimus can inhibit B cell differentiation *in vitro* (52), kidney transplant recipients with a B cell phenotype enriched for plasmablasts have a better transplant prognosis, i.e. better renal function and lower acute rejection incidence (53). In contrast, increased frequencies of B cells with a memory phenotype (CD27^+^CD38^+/-^) could be observed in the circulation of preeclamptic women (54, 55), suggesting that while the observed phenotypes could be beneficial for transplant survival they could be associated with the increased risk of complications in pregnant kidney transplant recipients.

Immunosuppressive drugs are able to cross the placenta and enter the fetal circulation (8, 32), thereby potentially influencing fetal immune development during pregnancy (33, 34) and influencing the offspring’s health in later life (56–58). We indeed observed that the neonatal immune system is affected at birth in offspring born to women with a kidney transplant. Paralleling our uterine and maternal blood results, Treg frequencies are decreased in cord blood of children born to kidney transplant recipients (22, 35–37, 39). Impaired Treg numbers and function at birth (cord blood) have been associated with an increased risk to develop sensitization to food allergens and atopic dermatitis in the first year of life (59, 60). We also observed decreased B cell frequencies in cord blood of neonates born to kidney transplant mothers, corresponding with prior reports (22, 35–38). Reduced B cell numbers at birth are associated with an increased hospitalization risk in the first year of life for children born from kidney transplant recipients (22, 61), and could potentially interfere with vaccination responses (62, 63). Next to the adaptive immune system, also the innate system is affected at birth in neonates born to kidney transplant recipients. Significantly decreased NKT-like cell and classical monocyte, and increased non-classical monocyte and intermediate monocyte percentages were observed in cord blood of infants born to kidney transplant recipients. A similar monocyte composition and NKT cell decrease can be found in inflammatory disorders such as systemic lupus erythematosus (SLE) and rheumatoid arthritis (64–67). As such, this pro-inflammatory monocyte state and altered NKT-like cell frequencies might predispose children of women with a transplanted kidney to develop inflammatory or autoimmune disorders. Indeed, SLE development is more common in children born to kidney transplant patients compared to controls (61). The longitudinal assessment of neonatal immunity in children born from women with a kidney transplant is an important consideration in order to fully understand long term effects of *in utero* exposure. Unfortunately, only few studies exist that indicate that immune changes can persist up to 1 year after birth (22, 62, 63, 68). Overall, the impaired neonatal innate and adaptive immune system at birth observed here underscores that children born to kidney transplant recipients could be at an increased risk for developing health complications early and later in life.

This study has certain limitations. Adverse pregnancy outcomes may be a result of several risk factors (5, 9–11, 16–19). Although the incidence of pre-eclampsia amongst our kidney transplant recipients was 35.7% (5 out of 14), corroborating reported incidence (3, 5–7), our sample size is unfortunately not sufficiently powered to allow for extensive covariate analysis. Delineating the effect of transplantation, immunosuppression, pre-pregnancy graft function, and so on, on the pregnancy-specific and neonatal immune system and correlating this with the development of adverse pregnancy outcomes, would require a larger and more diverse cohort. In addition, it would also be interesting to compare the pregnancy-specific and neonatal immune changes after other solid organ transplantations to find common or transplant-specific risk factors. Secondly, only changes in immune cell frequencies were reported here, as white blood cell counts were not available. However, frequency changes in immune cell subsets is likely to affect the immunological balance which in turn could affect pregnancy outcome.

In conclusion, the maternal peripheral, uterine, and neonatal immune system development is dysregulated in kidney transplant recipients, with distinct effects of the immunosuppressive drug regimen on frequency of HLA-DR^+^ Treg. This could have important consequences for the risk of pregnancy complications and health outcomes in the offspring. Moreover, it will be crucial to carefully select any immune intervention during pregnancy for its intended effect, placental accumulation, and possible side-effect on the neonatal immune system. Placental explants (69), uterine organoids (70, 71), and/or *ex vivo* placental perfusion experiments (72) will be important models to use during this decision-making process. Future studies that follow offspring’s health outcomes longitudinally, including vaccination responses and immunological evaluation at later time points, should be aimed at elucidating the effect of different drug regimens and dosages on pregnancy outcomes and the neonatal immune environment.

## Data Availability Statement

The raw data supporting the conclusions of this article will be made available by the authors, without undue reservation.

## Ethics Statement

The studies involving human participants were reviewed and approved by Commissie Mensgebonden Onderzoek region Arnhem-Nijmegen. The patients/participants provided their written informed consent to participate in this study.

## Author Contributions

Conceptualization: HH, OH, RM, IJ, and DF. Data curation: DF, LRM, RM, IJ, HH, and OH. Formal Analysis: DF and JG. Investigation/Data acquisition: DF, BC, TL, HH, OH, LM, MB, and GC. Methodology: DF, JG, and BC. Project administration: DF, OH, HH, RM, and IJ. Supervision: RM and IJ. Visualization: DF and JG. Writing – original draft: DF. Writing – review and editing: DF, JG, IJ, RM, and all authors. All authors contributed to the article and approved the submitted version.

## Conflict of Interest

The authors declare that the research was conducted in the absence of any commercial or financial relationships that could be construed as a potential conflict of interest.

## Publisher’s Note

All claims expressed in this article are solely those of the authors and do not necessarily represent those of their affiliated organizations, or those of the publisher, the editors and the reviewers. Any product that may be evaluated in this article, or claim that may be made by its manufacturer, is not guaranteed or endorsed by the publisher.
