# Supplementary material for: Maternal, Decidual, and Neonatal Lymphocyte Composition Is Affected in Pregnant Kidney Transplant Recipients
Source: Front Immunol. 2021 Oct 28;12:735564. doi: 10.3389/fimmu.2021.735564 (PMC8585145; doi:10.3389/fimmu.2021.735564)
Supplement: Supplementary file 1 [file DataSheet_1.pdf]

frontiers

Supplementary Material

Supplementary Table I List of antibodies used for multiparameter flow cytometry labeling.

**Surface antibodies**	**Clone**	**Source**
CD3-ECD	UCHT1	Beckman Coulter
CD3-PB	UCHT1	Beckman Coulter
CD3-PE	UCHT1	Beckman Coulter
CD4-AF700	RPA-T4	eBioscience
CD4-PC5.5	13B8.2	Beckman Coulter
CD8-APC-AF700	B9.11	Beckman Coulter
CD8-APC-AF750	B9.11	Beckman Coulter
CD8-ECD	SFCI21Thy2D3	Beckman Coulter
CD14-ECD	UCHT1	Beckman Coulter
CD16-FITC	3G8	Beckman Coulter
CD19-APC-AF750	J3-119	Beckman Coulter
CD24-APC	ALB9	Beckman Coulter
CD25-PC7	M-A251	BD Biosciences
CD27-PC5.5	1A4CD27	Beckman Coulter
CD38-PC7	LS198-4-3	Beckman Coulter
CD45-KO	J33	Beckman Coulter
CD45RA-FITC	ALB11	Beckman Coulter
CD45RA-ECD	2H4LDH11LDB9	Beckman Coulter
CD45RO-ECD	UCHL1	Beckman Coulter
CD56-APC	N901	Beckman Coulter
CD127-APC-AF700	R34.34	Beckman Coulter
CD197-BV421	G043H7	Sony
HLA-DR-PE	immu-357	Beckman Coulter
IgD-FITC	IADB6	Southern Biotech

**Intracellular antibodies**	**Clone**	**Source**
FoxP3-e450	PCH101	eBioscience
IFN-γ-PC7	4S.B3	eBioscience
IL-17-APC-AF780	eBio64DEC17	eBioscience
Rat serum		eBioscience

A - Decidua

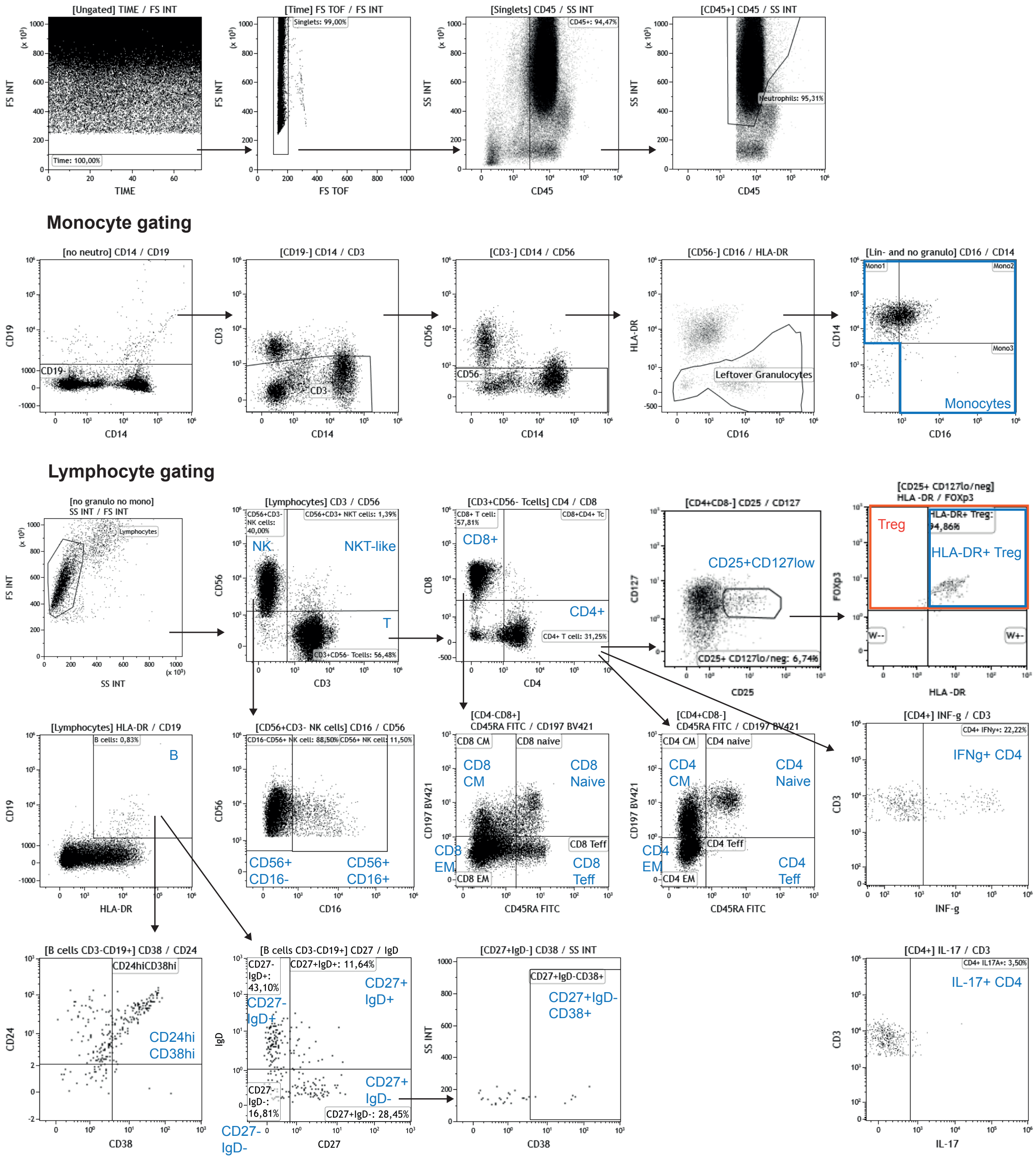
[Ungated] TIME / FS INT
Time: 100,00%
[Time] FS TOF / FS INT
Singlets: 99,00%
[Singlets] CD45 / SS INT
CD45+: 94,47%
[CD45+] CD45 / SS INT
Neutrophils: 95,31%
Monocyte gating
[no neutro] CD14 / CD19
CD19-
[CD19-] CD14 / CD3
CD3-
[CD3-] CD14 / CD56
CD56-
[CD56-] CD16 / HLA-DR
Leftover Granulocytes
[Lin- and no granulo] CD16 / CD14
Mono1
Mono2
Mono3
Monocytes
Lymphocyte gating
[no granulo no mono] SS INT / FS INT
Lymphocytes
[Lymphocytes] CD3 / CD56
CD56+CD3- NK cells: 40,00%
CD56+CD3+ NKT cells: 1,39%
NK
NKT-like
T
CD3+CD56- Tcells: 56,48%
[CD3+CD56- Tcells] CD4 / CD8
CD8+ T cell: 57,81%
CD8+CD4+ Tc
CD8+
CD4+
CD4+ T cell: 31,25%
[CD4+CD8-] CD25 / CD127
CD25+CD127low
CD25+ CD127lo/neg: 6,74%
[CD25+ CD127lo/neg]
HLA-DR / FOXp3
Treg
HLA-DR+ Treg: 94,86%
HLA-DR+ Treg
W--
W+-
[Lymphocytes] HLA-DR / CD19
B cells: 0,83%
B
[CD56+CD3- NK cells] CD16 / CD56
CD16-CD56+ NK cell: 88,50%
CD56+ NK cell: 11,50%
CD56+ CD16-
CD56+ CD16+
[CD4-CD8+] CD45RA FITC / CD197 BV421
CD8 CM
CD8 naive
CD8 Naive
CD8 EM
CD8 Teff
[CD4+CD8-] CD45RA FITC / CD197 BV421
CD4 CM
CD4 naive
CD4 Naive
CD4 EM
CD4 Teff
[CD4+] INF-g / CD3
CD4+ IFNy+: 22,22%
IFNg+ CD4
[B cells CD3-CD19+] CD38 / CD24
CD24hiCD38hi
CD24hi CD38hi
[B cells CD3-CD19+] CD27 / IgD
CD27- IgD+: 43,10%
CD27+IgD+: 11,64%
CD27+ IgD+
CD27- IgD+
CD27- IgD-: 16,81%
CD27+IgD-: 28,45%
CD27+ IgD-
CD27- IgD-
[CD27+IgD-] CD38 / SS INT
CD27+IgD-CD38+
[CD4+] IL-17 / CD3
CD4+ IL17A+: 3,50%
IL-17+ CD4

B - Maternal peripheral blood

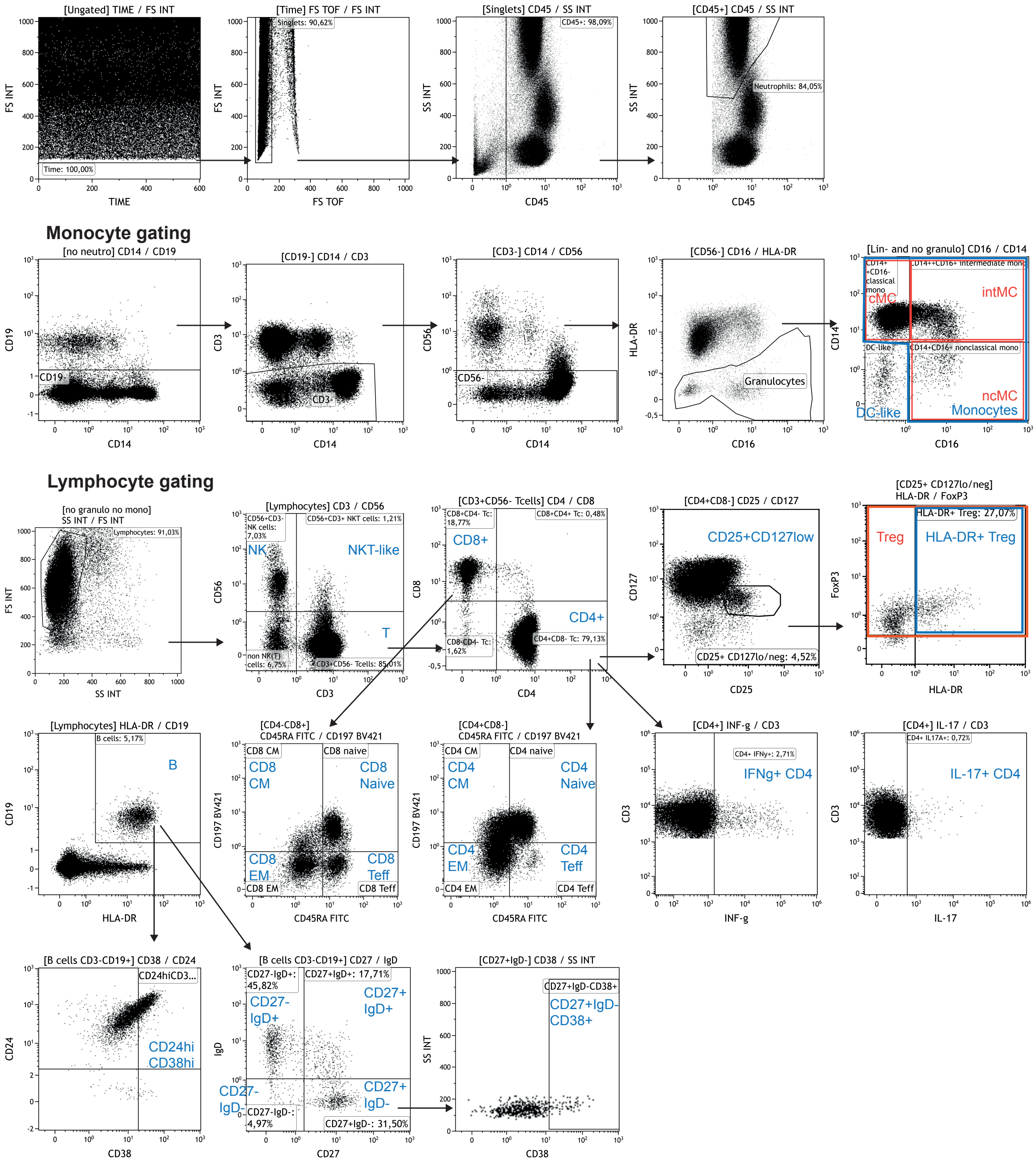

C - Cord blood

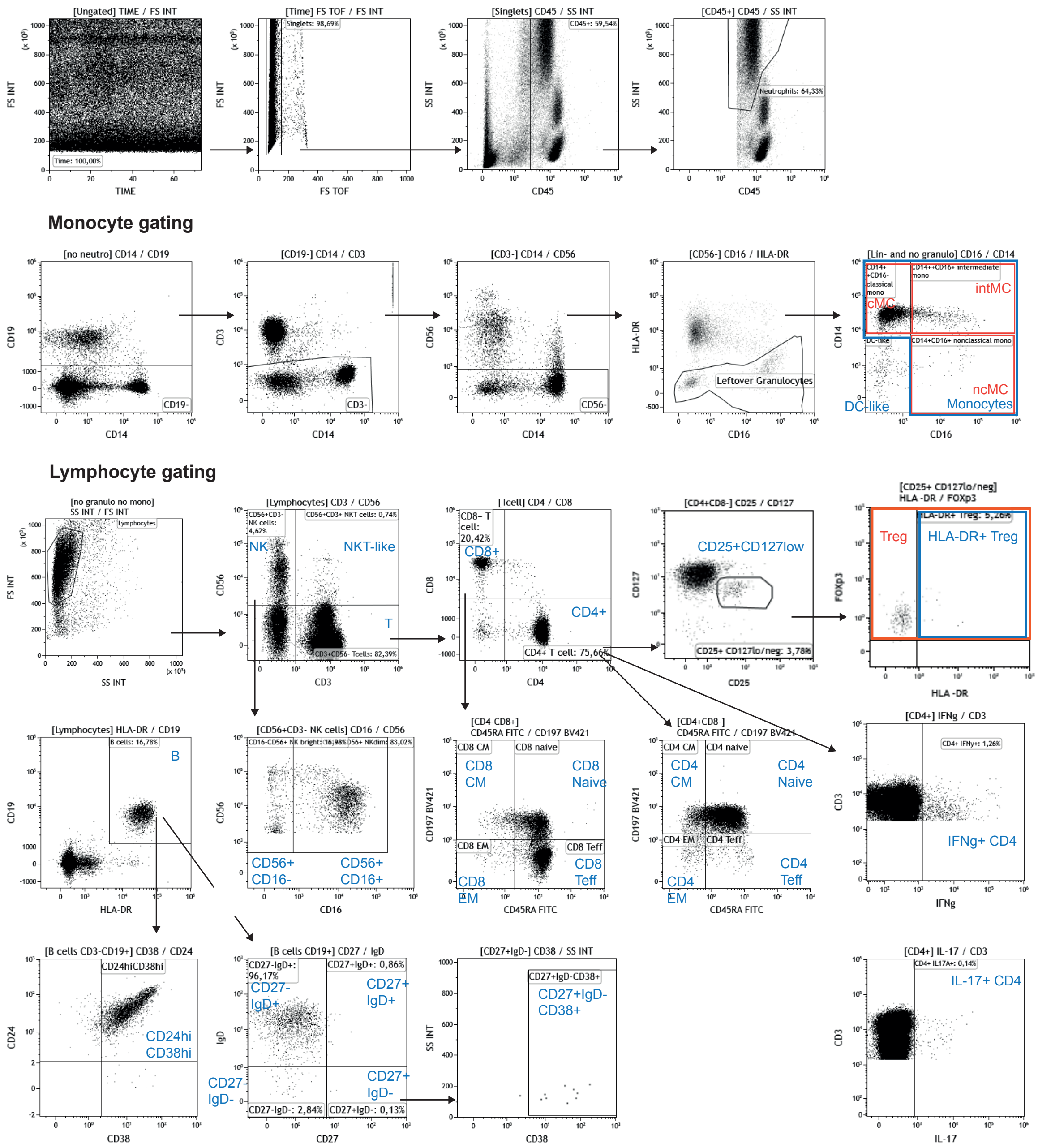

Supplementary Figure 1. Gating strategy. (**A**) Gating strategy and marker expression of a representative decidua sample, (**B**) a representative maternal peripheral blood sample, and (**C**) a representative cord blood sample. cMC = classical monocyte; intMC = intermediate monocyte; ncMC = non-classical monocyte.

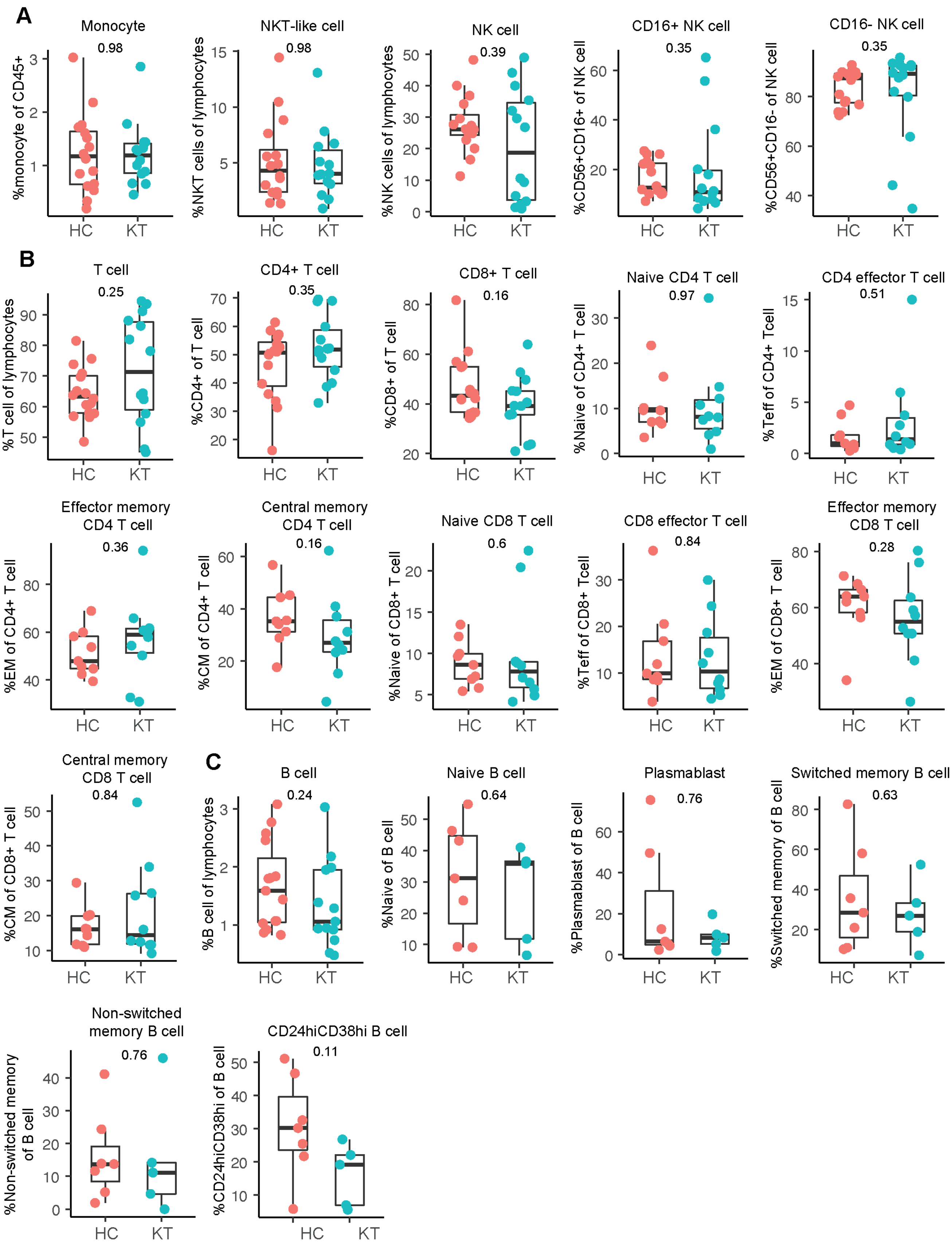
A
Monocyte
0.98
%monocyte of CD45+
NKT-like cell
0.98
%NKT cells of lymphocytes
NK cell
0.39
%NK cells of lymphocytes
CD16+ NK cell
0.35
%CD56+CD16+ of NK cell
CD16- NK cell
0.35
%CD56+CD16- of NK cell
B
T cell
0.25
%T cell of lymphocytes
CD4+ T cell
0.35
%CD4+ of T cell
CD8+ T cell
0.16
%CD8+ of T cell
Naive CD4 T cell
0.97
%Naive of CD4+ T cell
CD4 effector T cell
0.51
%Teff of CD4+ Tcell
Effector memory CD4 T cell
0.36
%EM of CD4+ T cell
Central memory CD4 T cell
0.16
%CM of CD4+ T cell
Naive CD8 T cell
0.6
%Naive of CD8+ T cell
CD8 effector T cell
0.84
%Teff of CD8+ Tcell
Effector memory CD8 T cell
0.28
%EM of CD8+ T cell
Central memory CD8 T cell
0.84
%CM of CD8+ T cell
C
B cell
0.24
%B cell of lymphocytes
Naive B cell
0.64
%Naive of B cell
Plasmablast
0.76
%Plasmablast of B cell
Switched memory B cell
0.63
%Switched memory of B cell
Non-switched memory B cell
0.76
%Non-switched memory of B cell
CD24hiCD38hi B cell
0.11
%CD24hiCD38hi of B cell
HC
KT

Supplementary Figure 2. Immune cell composition in decidua of kidney transplant patients (KT) and healthy individuals (HC). (**A**) Percentage of innate immune cells: monocytes, NKT-like cell, NK cell, $CD56^{+}CD16^{+}$ NK cell, and $CD56^{+}CD16^{-}$ NK cell; (**B**) percentage of T cell subsets: T cell, $CD4^{+}$, $CD8^{+}$, naïve ($CD45RA^{+}CCR7^{+}$), effector T cell (Teff; $CD45RA^{+}CCR7^{-}$), effector memory (EM; $CD45RA^{-}CCR7^{-)}$, and central memory (CM; $CD45RA^{-}CCR7^{+}$) CD4 and CD8 T cells; (**C**) and percentage of B cell subsets: B cell, naïve B cell ($CD27^{-}IgD^{+}$), plasmablast ($CD27^{+}IgD^{-}CD38^{+}$), switched memory ($CD27^{+}IgD^{-}$), non-switched memory ($CD27^{+}IgD^{+}$), and $CD24^{hi}CD38^{hi}$ B cell in decidua from KT and HC are shown.

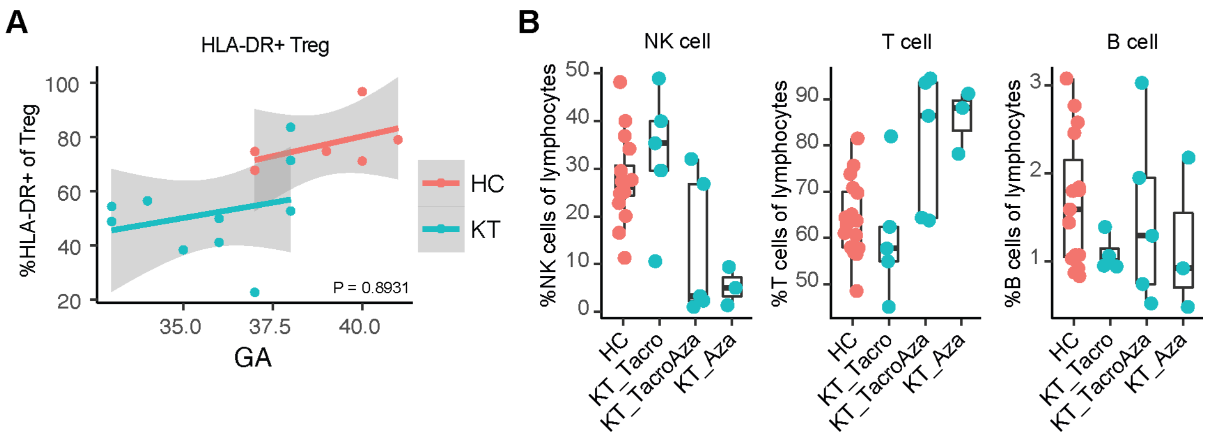

Supplementary Figure 3. Frequency of HLA-DR$^+$ regulatory T cells is affected in decidua of kidney transplant recipients (KT). (**A**) Percentage HLA-DR$^+$ Treg in decidua from KT and healthy individuals (HC) are plotted against gestational age (GA) at delivery. Lines indicate linear regression lines with the p-value indicating whether the slope of the regression lines is significantly different. (**B**) NK, T, and B cell frequencies separated based on use of tacrolimus (Tacro) or azathioprine (Aza).

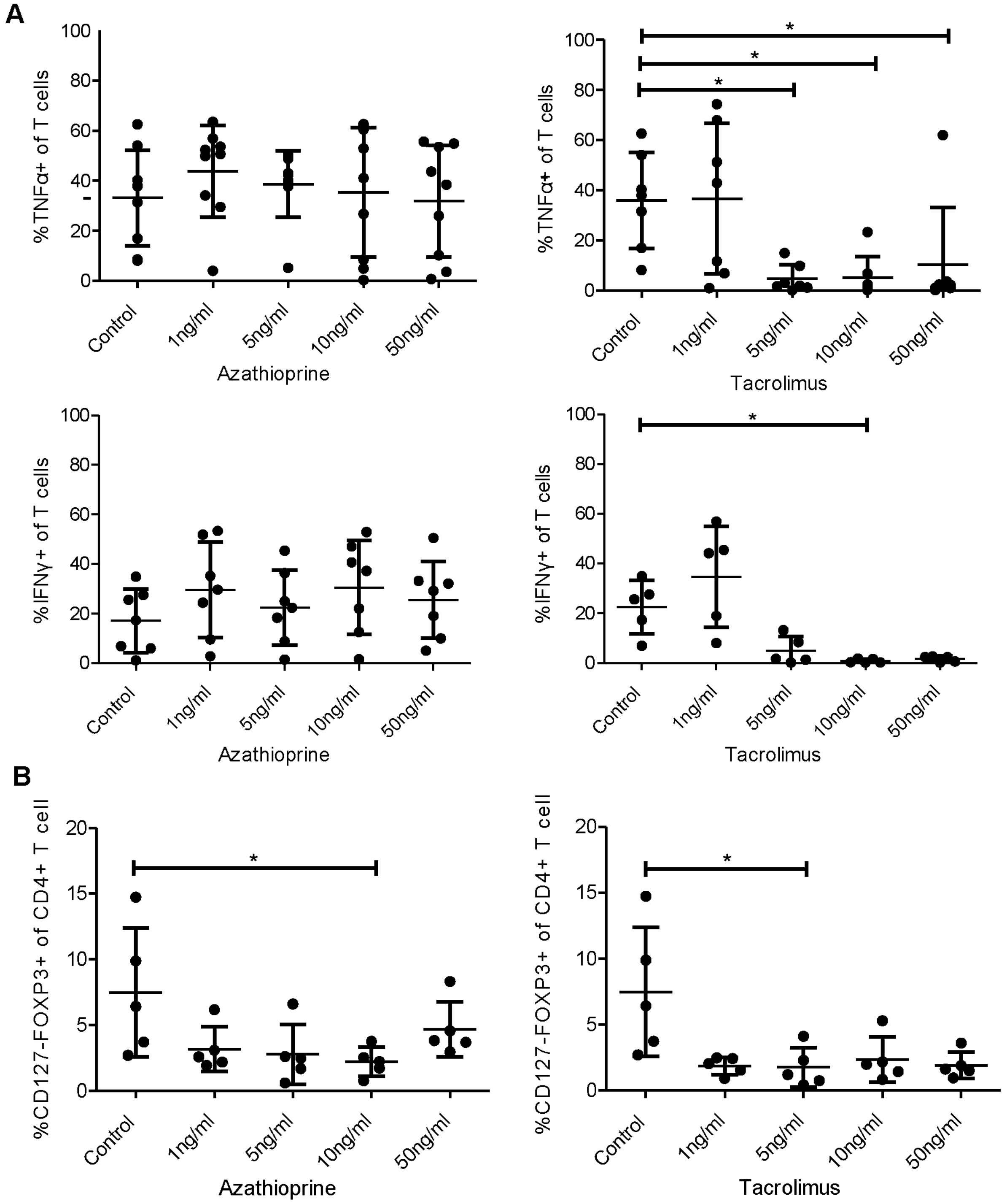

Supplementary Figure 4. In vitro effect of azathioprine and tacrolimus on first trimester decidual immune cell cytokine expression and regulatory T cell frequency. (**A**) Percentage of TNFα$^+$ and IFNγ$^+$ CD4$^+$ T cells, and (**B**) percentage CD127$^-$FOXP3$^+$ regulatory T cells after 5 days in vitro cell culture of first trimester decidual immune cells in the presence of azathioprine or tacrolimus. Mean ± SD are shown on the graph; * $p<0.05$.

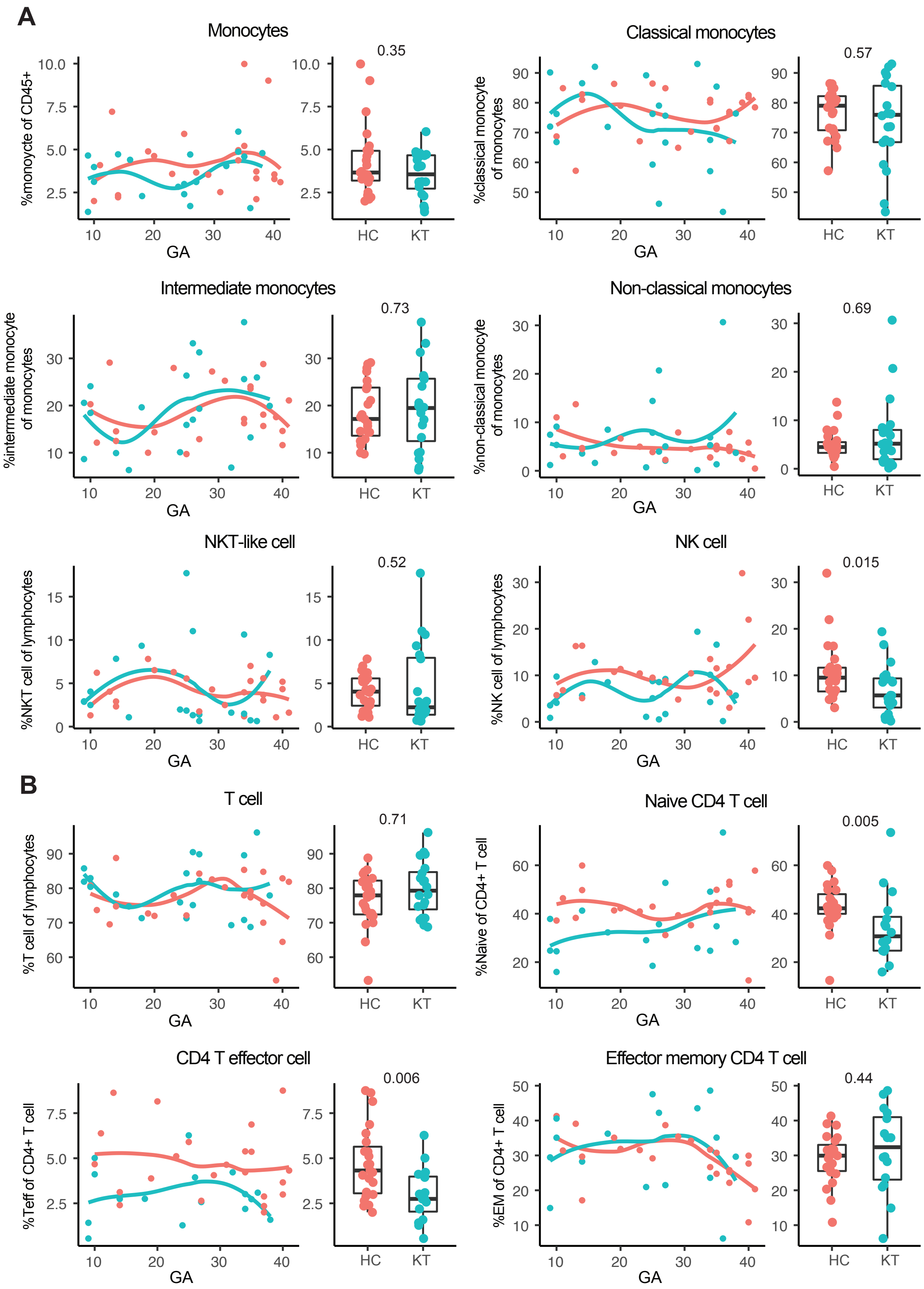
A
Monocytes
%monocyte of CD45+
GA
0.35
HC
KT
Classical monocytes
%classical monocyte of monocytes
0.57
Intermediate monocytes
%intermediate monocyte of monocytes
0.73
Non-classical monocytes
%non-classical monocyte of monocytes
0.69
NKT-like cell
%NKT cell of lymphocytes
0.52
NK cell
%NK cell of lymphocytes
0.015
B
T cell
%T cell of lymphocytes
0.71
Naive CD4 T cell
%Naive of CD4+ T cell
0.005
CD4 T effector cell
%Teff of CD4+ T cell
0.006
Effector memory CD4 T cell
%EM of CD4+ T cell
0.44

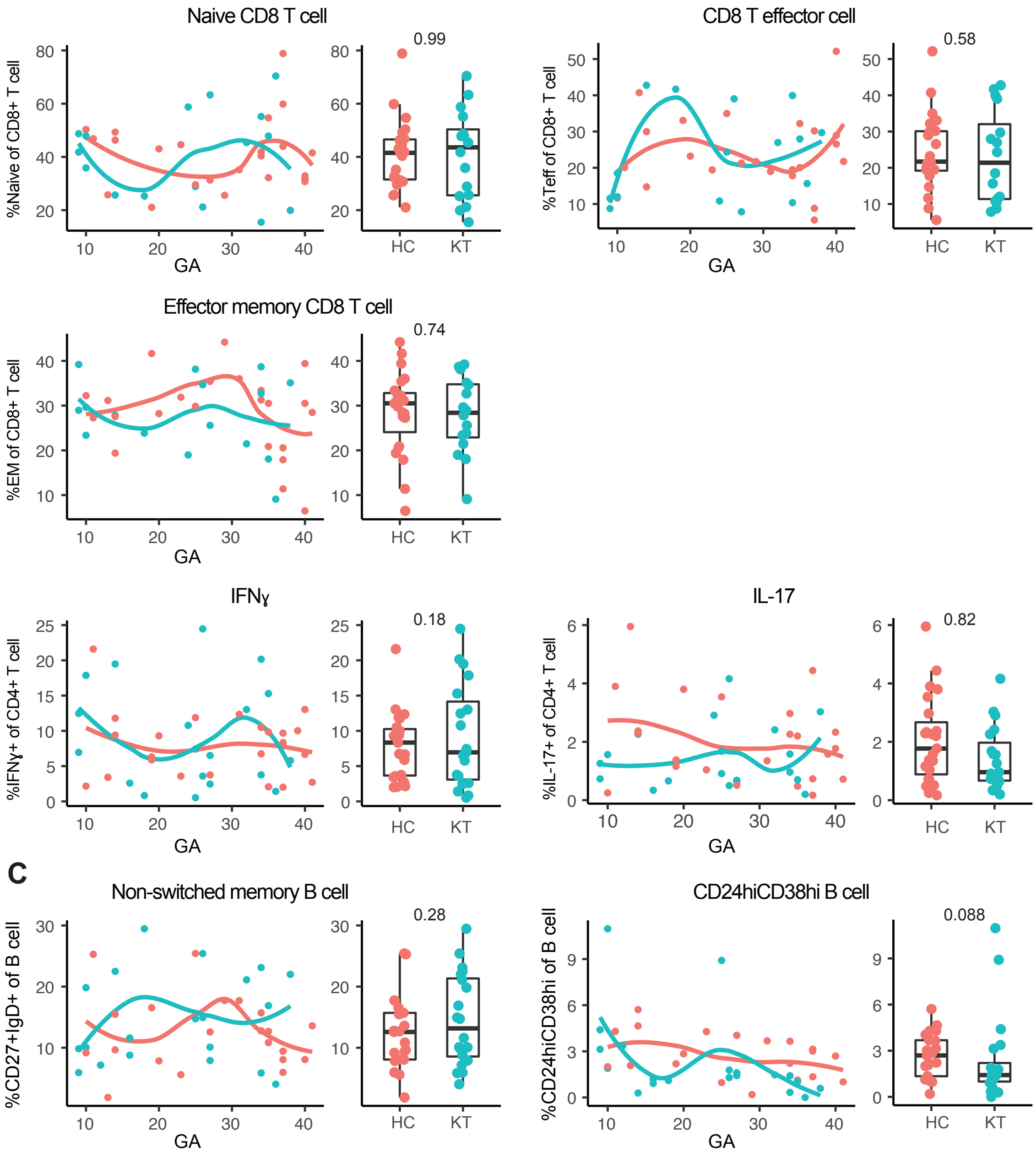

Supplementary Figure 5. Maternal systemic immunity is affected in pregnant kidney transplant recipients (KT). (**A**) Percentage of innate immune cells: monocytes, classical, intermediate, and non-classical monocytes, NKT-like cell, and NK cell; (**B**) percentage of T cell subsets: T cell, naïve ($CD45RA^+CCR7^+$), effector T cell (Teff; $CD45RA^+CCR7^-$), effector memory (EM; $CD45RA^-CCR7^-$) CD4 and CD8 T cells, IFN-γ^+, and IL-17^+ $CD4^+$ T cells; (**C**) and percentage of B cell subsets: non-switched memory ($CD27^+IgD^+$) and $CD24^{hi}CD38^{hi}$ B cell in peripheral blood from KT and healthy individuals (HC) are shown. Frequencies of peripheral blood immune cells are depicted both in boxplots (median + interquartile range) and as regression (LOESS) with gestational age (GA) at time of sample collection.

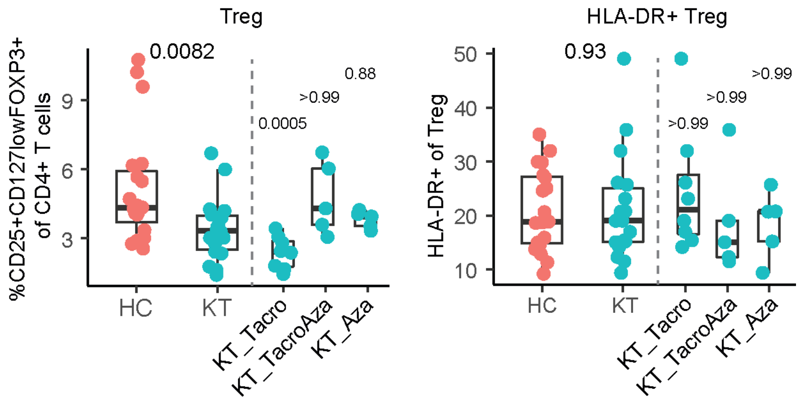

Supplementary Figure 6. Frequency of regulatory T cells in peripheral blood of kidney transplant recipients (KT) is primarily affect by tacrolimus use. Percentage regulatory T cells (Treg) and HLA-DR^{+} Treg in peripheral blood from pregnant KT and healthy individuals (HC), separated based on which combination of tacrolimus (Tacro) and azathioprine (Aza) is used.

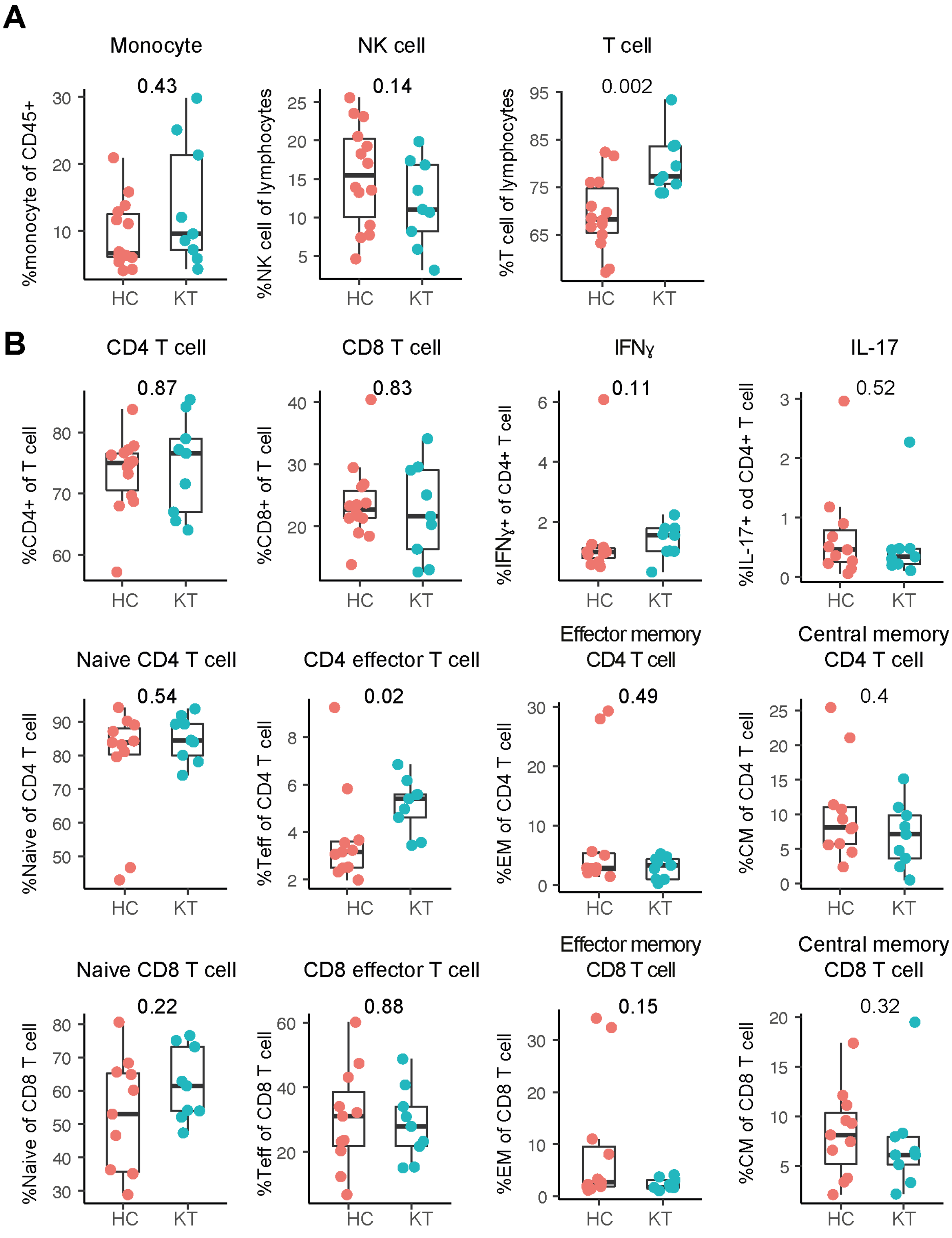

Supplementary Figure 7. Innate immunity is affected in neonates born to pregnant kidney transplant recipients (KT). (**A**) Percentage of monocytes, NK cell, and T cell; (**B**) percentage of T cell subsets: $CD4^+$, $CD8^+$, IFN-γ^+ $CD4^+$ T cell, IL-17^+ $CD4^+$ T cell, naïve ($CD45RA^+CCR7^+$), effector T cell (Teff; $CD45RA^+CCR7^-$), effector memory (EM; $CD45RA^-CCR7^-$), and central memory (CM;

CD45RA$^-$CCR7$^+$) CD4 and CD8 T cells in cord blood of neonates born to KT and healthy individuals (HC) are shown.

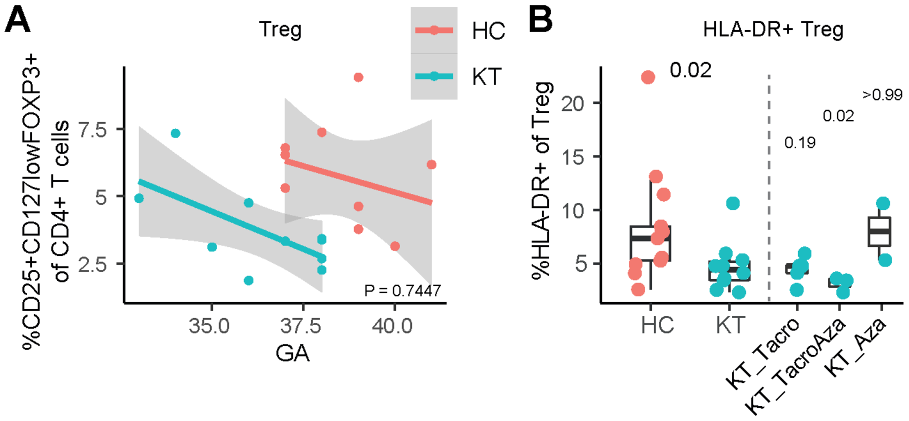

Supplementary Figure 8. HLA-DR^{+} Treg in neonates born to pregnant kidney transplant recipients (KT) are primarily affected by exposure to tacrolimus. (**A**) Percentage of regulatory T cell (Treg) in cord blood of neonates born to KT and healthy individuals (HC) are plotted against gestational age (GA) at delivery. Lines indicate linear regression lines with the p-value indicating whether the slope of the regression lines is significantly different. (**B**) Percentage HLA-DR^{+} regulatory T cell (Treg) in cord blood of neonates born to KT and HC are shown, separated based on which combination of tacrolimus (Tacro) and azathioprine (Aza) was used.
